# A national cohort study of spiritual and religious practices of older people with intellectual disability

**DOI:** 10.1177/17446295231163977

**Published:** 2023-03-23

**Authors:** Fiona Timmins, Darren McCausland, Damien Brennan, Fintan Sheerin, Retha Luus, Philip McCallion, Mary McCarron

**Affiliations:** Midwifery and Health Systems, School of Nursing, 8797 UCD, Dublin, Ireland; Trinity Centre for Ageing and Intellectual Disability, Trinity College, 8809 The University of Dublin, Dublin, Ireland; The School of Nursing and Midwifery, 8809 Trinity College, Dublin, Ireland; Trinity Centre for Ageing and Intellectual Disability, Trinity College, 8809 The University of Dublin, Dublin, Ireland; The School of Nursing and Midwifery, 8809 Trinity College, Dublin, Ireland; Trinity Centre for Ageing and Intellectual Disability, Trinity College, 8809 The University of Dublin, Dublin, Ireland; The School of Nursing and Midwifery, 8809 Trinity College, Dublin, Ireland; Trinity Centre for Ageing and Intellectual Disability, Trinity College, 8809 The University of Dublin, Dublin, Ireland; The School of Nursing and Midwifery, 8809 Trinity College, Dublin, Ireland; The School of Nursing and Midwifery, 8809 Trinity College, Dublin, Ireland; Temple School of Social Work, College of Public Health, 6558 Temple University, Philadelphia, PA, USA; Trinity Centre for Ageing and Intellectual Disability, Trinity College, 8809 The University of Dublin, Dublin, Ireland; The School of Nursing and Midwifery, 8809 Trinity College, Dublin, Ireland

**Keywords:** intellectual disability, older people, spirituality, health

## Abstract

**Background:** Spirituality and spiritual support for older people with intellectual disability are deemed important, however little is known about their specific needs. This paper reports for the first time on the religious and spiritual practices of older adults with intellectual disability. **Methods:** A national longitudinal study examined the prevalence of spiritual practices among older people with intellectual disability in the Republic of Ireland. **Results:** Older people with intellectual disability seek and receive solace from religious and spiritual practices, especially if they are lonely, in poor health, distressed or bereaved. There is likely a social benefit to spiritual and religious aspects of life that would be beneficial to explore further. **Conclusions:** Globally more research is required and efforts should be made to ensure greater opportunities for inclusion in societal spiritual and religious activities and to more clearly determine the spiritual needs of this population.

## Introduction

Despite an emerging concern with end of life care, mental health and social support needs of people with intellectual disability, little attention is paid to their spiritual needs. Defining spirituality is complex and multidimensional, and whilst there is no universally agreed definition, one recent project aimed at improving spiritual care competence in healthcare (EPICC) (2021) explains spirituality as:

“The dynamic dimension of human life that relates to the way persons (individual and community) experience, express and/or seek meaning, purpose and transcendence, and the way they connect to the moment, to self, to others, to nature, to the significant and/or the sacred.” (Kleiven et al., 2021)

For some, spirituality involves an adherence to a religion, or a set of religious beliefs and values, while for others these beliefs and values are spiritual but not religious (Weathers et al., 2016). Historically religion has been a core feature of the social fabric and care systems in the Republic of Ireland (ROI) (Timmins 2013), and whilst there is a growing perception of secularity across Europe, the most recent Irish census (CSO 2016) showed much of the population still had an affiliation with Christianity, with most (78.3%) identifying as Roman Catholic. Interestingly most health care sites engage the services of a Healthcare Chaplain (Timmins et al., 2017). While church attendance is notably on the decline, current longitudinal data on aging in the ROI indicates church engagement by older people with consequential positive associations with health and wellbeing (Orr et al 2019).

While supporting spiritual and religious practices in health and social care settings is acknowledged as a universal human right internationally, there is limited consistent advice for the acknowledgement and support of spiritual needs for people with intellectual disability within the institutionalised context or within state supported community services in the ROI. Indeed spirituality and spiritual support receive very little attention among this population (McCarron et al., 2011). While people with intellectual disability are increasingly supported by families or in state supported community settings, there is still a large presence of institutionalised services people with intellectual disability (most with community links) in the ROI. Many of these services have historical religious origins and many have the support of a Healthcare Chaplain (Powell 2019).

## Background

While spirituality is acknowledged as a component of holistic understanding of people with intellectual disability (Tencza and Forsythe 2021) with many agreeing that distinct spiritual needs exist, there is disagreement, confusion and often reluctance to provide spiritual care in formal institutionalised or community care settings (Crotty and Doody 2016). Büssing (2021) broadly explains that spiritual care is provided when a person’s struggles, fears and worries are listened to and their spiritual needs and underlying spirituality, whatever this may mean to them, are supported. For staff who provide social support and care, while many will provide spiritual support in keeping with their own faith, the requirements of the organisation or prompts from the individual, overall there is a lack of clear national guidance for staff who support those attending services for people with intellectual disability (Sango and Forrester-Jones 2014) resulting in gaps and omissions. One further concern relates to cognitive capacity to understand such concepts. Some definitions of spirituality purport that full cognitive ability is required for a person to be spiritual (Weathers et al., 2016). However this is a subject of debate (Timmins et al 2016), and certainly Healthcare Chaplains working in institutionalised settings for those with intellectual disability believe that spirituality and spiritual care are important, especially at time of loss and grief (Powell 2019).

However there is an acknowledged lack of national guidance for the spiritual support of people with intellectual disability, in the UK for example (Sango and Forrester-Jones 2014). Yet one international consensus statement for end of life care highlighted the importance of spirituality for this cohort (McCallion et al., 2017). However beyond this there is little empirical research on this topic. Early qualitative research with nurses working alongside people with intellectual disability revealed that they believed that spiritual needs existed and developed an awareness of this based on the person’s religious background and verbal cues (Narayanasamy et al 2002). Interventions, which were often described as “procedural” (such as attendance at religious services and providing religious support) appeared to support clients’ needs (Narayanasamy et al 2002:954). Before this time there was little research or understanding in this field, and this author and others such as Swinton (2004) began to suggest that people with intellectual disabilities “need to be given accessible information and opportunities in order that they can make informed spiritual choices” and understood that health care workers were ill equipped for this task. Swinton’s (2004) interviews revealed that some people with intellectual disabilities expressed their spirituality through religion, whereas for others this related to meaning and connectedness. At the same time faith communities were found to have practices that were often perceived as exclusionary (Swinton 2004). Yet support to attend faith based services along with other community activities is encouraged through national policy in the UK but few guidelines exist for practice (Sango and Forrester-Jones 2014).

Spirituality is concerned with how a person finds meaning, purpose and connection in life (Weathers et al., 2016). Spirituality may be distinguished from religion, but for some spirituality may involve mainstream or personalised/adapted religious beliefs and practices. These understandings of spirituality are mirrored within intellectual disability literature (Sango and Forrester-Jones 2019, Glicksman 2011, Swinton 2004, Narayanasamy et al., 2002). However, beyond anecdotal accounts (Powell 2019) little empirical evidence exists that describes the spiritual beliefs, activities or needs of older people with intellectual disability. There is some evidence that spirituality can have supportive benefits for family carers in understanding and making sense of supporting others with disability (Karaca and Konuk Şener 2021). However overall, there is a dearth of literature on this topic within this population, and the sparse literature that exists mostly reflects research that is more than a decade old.

In 2007 for example, Gratsa et al. found that spirituality was a core component of the support required by those with intellectual disability (aged between 35 and 65) and their family carers. In 2011 Bertelli et al. found the lowest scores in the area of spiritual wellbeing when examining quality of life of younger people with intellectual disability (maximum age 50 years). Beyond this there is very little research available to provide an understanding of spiritual practices, beliefs, understandings or needs of this cohort. This paper therefore uses a large dataset to systematically analyse spiritual practices among older people with intellectual disability in the ROI.

## Purpose

This study aims is to describe spiritual practices in older people with intellectual disability in the Republic of Ireland (ROI).

### Objectives

To examine spiritual practices among older people with intellectual disability using a robust national longitudinal dataset.

### Design

The design for this study is a prospective cohort study, named the Intellectual Disability Supplement (IDS) of the Irish Longitudinal Study on Aging (TILDA), IDS-TILDA (McCarron & McCallion 2016). This dataset is the world’s only longitudinal study on the health and well-being of older adults with an intellectual disability. This is a unique study internationally that investigates the health and well-being of people with intellectual disability in the ROI during 3-yearly cycles. It is linked to a national general population study of ageing, in this case the Irish Longitudinal Study on Ageing (TILDA) (Kenny et al., 2010). The sample for the IDS-TILDA is drawn randomly from the National Ability Supports System (NASS). The NASS is managed by the Irish Health Research Board and collects information on all people in the ROI with an intellectual disability who receive services from the state (Health Research Board, 2023). This database provides objective up-to-date information about all people in Ireland who have been diagnosed with an intellectual disability and who are in receipt of support services (Hourigan et al., 2017). This database is unique and no such systematic data collection regarding people with intellectual disability exists across Europe.

The database at study commencement had 26,066 participants of all levels of disability, and across all living circumstances (McCarron et al., 2011). Recruitment of participants to the IDS-TILDA study, from this dataset, commenced in 2009 and details on the recruitment, returns and procedures for wave 3 are described in McCarron et al. (2017). Now in its fourth wave, IDS TILDA aims to identify health and social behaviour and identify disparities among older adults with intellectual disability. Wave 3 data (collected in 2015-2017, McCarron et al 2017) informed this paper. It represents a remaining sample (n = 609, 86% retention) from the original wave 1 sample (n = 753) drawn randomly from the NASS. This reflects a nationally representative sample (n = 609) that includes adults aged 40 years and older with intellectual disability in the ROI.

The overarching aims of IDS-TILDA are:

• To understand the health characteristics of people ageing with an intellectual disability;
• To examine the service needs and health service utilization of people ageing with an intellectual disability;
• To identify disparities in the health status of adults with an intellectual disability as compared to TILDA findings for the general population; and
• To support evidence-informed policies, practices and evaluation.

In wave 3 a large range of data are collected relating to demographic profiles, chronic conditions, social activity, family and social networks, loneliness and living circumstances, described in more detail in McCarron et al. (2017). For example quality of life is assessed using the *Quality of life sub-scale* from the Personal Wellbeing Index-Intellectual Disability Version (Cummins, 1997). This is an eight-item scale with responses on an 11-point rating scale. Two subscales measure global life satisfaction, the *Satisfaction with Life Scale* (SWLS), a 5-item self-report scale rated on a 6-point scale and the *Purpose in Life Questionnaire* is a 7-item subscale from The Ryff Psychological Wellbeing Scale (Ryff, 1989), rated on a 6-point scale. Data is also collected related to causes of distress, religion and sources of hope, peace or comfort. The components listed in Table 24, taken from these aforementioned measures, are combined to form personal wellbeing index (PWI) which provides relevant data about spiritual and religious practices, and their relationship to quality of life.

### Sample

Participants were originally selected for the first wave in 2007. A random selection of people over the age of 40 was identified from within the NASS database. Using a gatekeeper (the regional database controllers), invitation packs were sent to potential participants. The same participants were involved in subsequent waves (2, 3 and 4) and the same cohort were invited to continue their participation in the study. Of the 609 participants who took part in wave 3 (2015-2017) 323 were included for the purposes of this paper, as this was the number that were able to complete the required elements of the questionnaire. The questionnaire was only completed if participants could self-report or self-report with proxy assistance. As such, responses from participants that did not meet this requirement or complete the required elements of the survey were removed from the analysis. This is therefore a full representative sample, as those that did not complete were cognitively unable to do so.

### Data collection

Data collection for wave 3 IDS TILDA comprised completion of a pre-interview questionnaire, posted to participants a minimum of one week prior to a face to face interview. This collected information related to health conditions, mental health, health service usage/interactions and prescribed medication (McCarron et al 2017). Participants, or their advocate/proxy then attended a face to face interview where a range of IDS TILDA data were collected. These were collected using bespoke computer assisted software, termed computer assisted personal interview (CAPI). The CAPI provides for valid and reliable measurement of cognitive, behavioural psychological health, behavioural health, physical activity, functional limitations and activities of daily living. Select data relating to spiritual and religious practices, including membership of organisations, clubs or societies and social activity settings were used to inform this paper.

### Ethical Approval

The Local Faculty Ethical Committee, and the 138 service providers involved, approved all four waves of this study.

### Data analysis

RStudio version 1.2.5033 was used to analyse the collected data. After considering descriptive analyses, a set of variables (predictors) were identified. Associations between these, and the following dependent variables, were explored within the dataset:

• Things that give hope, peace or comfort
• Activities during difficult times
• What helps to feel at peace and at ease in life

The descriptive and bivariate analyses were followed by a stepwise logistic regression where a combination of forward selection and backward elimination was used to perform variable selection. The output reported from the logistic regression model includes the odds ratio, 95% confidence interval of the odds ratio and p-value of each variable selected by the procedure.

## Results

Most participants were female (n=181, 56%) aged between 50 and 64 years old (n=201, 62.2%) (Table 1). Levels of intellectual disability varied, and many participants lived in group community home settings (n=157, 48.6%) (Table 1). Almost a third (n=95, 31%) of the participants had access to 24-hour support from nursing staff within their residence.

**Table 1.** Demographic profile.

		*n* (%)
Gender	Male	142 (44%)
	Female	181 (56%)
Age	<50	38 (11.8%)
	50-64	201 (62.2%)
	65+	84 (26%)
Aetiology of ID	Down Syndrome	49 (15.2%)
	Unknown/Other/Don't know/Missing	274 (84.8%)
Level of ID	Mild	117 (40.3%)
	Moderate	157 (54.1%)
	Severe/Profound	16 (5.5%)
Residence	Independent/Family	81 (25.1%)
	Community group home	157 (48.6%)
	Residential care	85 (26.3%)

Participants were asked about their religion. For those who answered this question (n=288, 89.2%) most of these participants (n=215, 74.7%) indicated that religion was important in their life while the majority (n=231, 80.8%) indicated that religion gave them strength and comfort. Table 2 shows participants’ membership of certain organisations, clubs or societies.

**Table 2.** Membership of organisations, clubs or societies.

	No	Yes
Church or religious groups	304 (94.1%)	19 (5.9%)
Charitable associations (e.g. St Vincent De Paul’s)	311 (96.3%)	12 (3.7%)
Arts or music	275 (85.1%)	48 (14.9%)
Special Olympics Network	269 (83.3%)	54 (16.7%)
Arch Club	306 (94.7%)	17 (5.3%)
Advocacy Group	277 (85.8%)	46 (14.2%)
Social, Sports or Leisure club	264 (81.7%)	59 (18.3%)

With regards to church or religious groups, only 5.9% indicated that they were active members. For those that participated in this way their means of participation varied (Table 3).

**Table 3.** Social activity setting.

	Within Community Setting	Within ID Service	Both within community & ID
Church or religious groups	16 (88.9%)	2 (11.1%)	0 (0.0%)
Charitable associations	9 (81.8%)	1 (9.1%)	1 (9.1%)

Participants were also asked about what gives them hope, peace or comfort. Of these 190 (58.8%) indicated that talking to staff supported this, while 49.8% experience this when talking to friends or family; 45.2% when listening to music; 37.2% when going for a walk and 35% when praying (Table 4). Participants were asked which of these aforementioned items they would do most often during the difficult times. Participants were also asked what helps them to feel peace and at ease in life (Table 5) (322 responses).

**Table 4.** Which of the items related to hope, peace or comfort would you do most often during the difficult times?

	*n* (%)
Talking to friends/family	68 (22.2%)
Talking to staff	102 (33.3%)
Praying	15 (4.9%)
Going to a religious/faith-based service	14 (4.6%)
Spending quiet time on my own	27 (8.8%)
Listening to music	24 (7.8%)
Going for a walk	29 (9.5%)
Meditating/yoga/other practice	0 (0%)
Being in nature	3 (1%)
Other	24 (7.8%)

**Table 5.** What helps you to feel peace and at ease in your life?

	No	Yes
Talking to friends/family	201 (62.4%)	121 (37.6%)
Talking to staff	204 (63.4%)	118 (36.6%)
Praying	258 (80.1%)	64 (19.9%)
Going to a religious/faith-based service	268 (83.2%)	54 (16.8%)
Spending quiet time on my own	245 (76.1%)	77 (23.9%)
Listening to music	209 (64.9%)	113 (35.1%)
Going for a walk	243 (75.5%)	79 (24.5%)
Being in nature	303 (94.1%)	19 (5.9%)
Meditating/yoga/other practice	317 (98.4%)	5 (1.6%)
Other (Please specify)	260 (80.7%)	62 (19.3%)

Cummins (1997) personal well-being Index, designed for use in intellectual disability populations was used to explore elements of participants’ quality of life (Table 6, response rate 96.9%, n=313). Items included in this tool include standard of living, health, achieving in life, relationships, safety, community-connectedness, and future security. One additional item was added to this index (item 1, Table 6) and each item provided valuable data relating to spirituality with relation to its defining features. These participants generally reported positive well-being, and the majority reported positive on the item for community-connectedness (89.4%, n=277) , and the majority of these (75%, n=113) had high overall scores on the personal wellbeing index (PWI) (i.e., scores greater than 78.6). Few expressed worries (Table 7), and life events did not seem to cause distress (Table 8).

**Table 6.** Components of Personal Wellbeing Index (Cummins & Lau 2005).

	Sad	Neither happy nor sad	Happy
How happy do you feel about your life as a whole?	5 (1.6%)	25 (8%)	283 (90.4%)
Like the money you have and the things you have and the things you own?	3 (1%)	23 (7.3%)	287 (91.7%)
How healthy you are?	7 (2.3%)	30 (9.6%)	274 (88.1%)
The things you make or the things you learn?	2 (0.6%)	38 (12.3%)	270 (87.1%)
Getting on with the people you know?	2 (0.6%)	24 (7.7%)	284 (91.6%)
How safe you feel?	4 (1.3%)	23 (7.4%)	283 (91.3%)
Doing things outside your home?	8 (2.6%)	25 (8.1%)	277 (89.4%)
How things will be later on in your life?	9 (2.9%)	108 (35.2%)	190 (61.9%)

**Table 7.** Do you worry …?

	No	Sometimes	A lot
Do you worry about your family or friends?	143 (51.3%)	101 (36.2%)	35 (12.5%)
Do you worry about the future?	199 (71.6%)	60 (21.6%)	19 (6.8%)
Do you worry that something bad will happen?	212 (76.3%)	52 (18.7%)	14 (5%)
Do you worry about what you are doing tomorrow?	225 (80.9%)	38 (13.7%)	15 (5.4%)
Can you stop yourself worrying?	125 (45%)	99 (35.6%)	54 (19.4%)
Do you worry about dying?	238 (85.6%)	29 (10.4%)	11 (4%)

**Table 8.** Life events that cause distress.

	Yes	No
Change at or from work or day service	40 (12.7%)	274 (87.3%)
Death of a parent	12 (3.8%)	302 (96.2%)
Death of a sibling	15 (4.8%)	299 (95.2%)
Death of other relative	24 (7.6%)	290 (92.4%)
Death of a friend	77 (24.5%)	237 (75.5%)
Death of a pet	7 (2.2%)	307 (97.8%)
Major illness of a relative, caregiver or friend	43 (13.7%)	271 (86.3%)
Death of a significant other (other than a relative, caregiver or friend)	9 (2.9%)	305 (97.1%)
Moving within service organisation	17 (5.4%)	297 (94.6%)
Moving from his/her family home to a service supported home	2 (0.6%)	312 (99.4%)
Change in frequency of visits from or to family / friend	25 (8%)	289 (92%)
Major illness or injury	24 (7.6%)	290 (92.4%)
Break up of a steady relationship / Divorce	2 (0.6%)	312 (99.4%)

Questions relating to quality of life captured aspects of participants spiritual care needs with a focus on their surrounding supports and the influence this had on spiritual wellbeing. 320 participants responded to questions related to quality of life, and the majority of these scored highly (n=290, 90.6%). In terms of loneliness almost one half reported feeling lonely (n=131, 42.7%). Although most of these respondents (n=295, 95.8%) indicated that they had someone to confide in, either a sibling (n=92, 31.2%) or a friend (n=72, 24.4%). The majority indicated that they had a sister (n=247, 76.5%) or brother (n=237, 73.4%) while almost half (n=187, 57.9%) had nieces or nephews. Less (n=65, 20.1%) reported having a mother or father (n=24, 7.4%). The majority (n=228, 72%) reported frequent and regular family contact, with many of these (n=106, 34.6%) indicating that this was once or twice a week. However, many participants also indicated difficulty participating in social activities outside of their home (n=126, 40.4%). Of these respondents 60 (47.6%) indicated that this was due to health considerations or that they were physically unable. Many (n=94, 74.6%) were dependent on the assistance of others. Other reasons (such as transport or financial means) did not rate highly as barriers.

In relation to going to a religious/faith-based service, logistic regression modelling revealed some significant associations. Those who attended church or religious group were more likely to receive comfort from this (p=0.028). Those who felt distressed by the death of a friend, found it difficult to make friends, or who self-reported physical health as fair/poor or felt less happy about life, were more likely to receive comfort from going to a religious/faith-based service (p<0.05). At the same time, difficulty making friends (p=0.031); major illness or injury (p=0.004); poor/fair physical health (p=0.004) and feeling less happy about life (p=0.021) were more likely to predict attendance at religious/faith-based services during difficult times. People with intellectual disability were also more likely to attend religious/faith-based services during difficult times if they lived in community group homes (p=0.003) or residential care (p=0.02). Having moderate disability (p=0.002) and living in a community group home (p=0.008) were significantly associated with feeling peace and at ease in life after going to a religious/faith-based service.

With regard to prayer, factors associated with feeling peace and at ease in life after praying include female gender (p=0.028); living in community group home (p=0.04); death of relative (p=0.042); feeling lonely (p=0.005) and poor physical health (p=0.015) (Table 9). In terms of items that bring hope, peace or comfort (such as praying or going to a religious/faith-based service) only self-reported physical health was found to be significantly related to praying (p=0.003). Those that self-reported their physical health as fair, or poor were more likely to receive hope, peace, or comfort from praying.

**Table 9.** Factors associated with feeling peace and at ease in life after praying (reduced model).

Factor	Odds Ratio	2.5 %	97.5 %	P-value
Gender	2.4	1.12	5.42	**0.028**
Moderate ID	0.857	0.406	1.81	0.684
Severe/Profound ID	0		4.26 × 10^28^	0.987
Community group home	0.386	0.152	0.955	**0.04**
Residential care	0.554	0.197	1.49	0.249
Death of other relative	3.8	1	13.8	**0.042**
Major illness or injury	2.95	0.707	12	0.129
Do you ever feel lonely?: Yes	3.1	1.42	7.02	**0.005**
Physical health: at least good	0.239	0.073	0.759	**0.015**
How things will be later on in your life?: Happy	1.86	0.845	4.37	0.135

## Discussion

This study reports the first large scale findings about the importance of spirituality for older people with intellectual disability. Our findings confirm recent qualitative findings that spirituality serves an important function for this population (Thompson et al., 2020, Sango and Forrester-Jones 2018, Dew et al., 2019). This study also highlights for the first time those aspects of spiritualty and religious rituals that older adults with intellectual disability find important. This study is novel insofar as it adds important information to the sparse knowledge base in the field.

In keeping with Sango and Forrester-Jones’s (2017) systematic review findings, participants exhibited a spiritual and/or religious identity through their reported behaviours. Firstly, religious, and spiritual practices were important aspects of the life of many participants that gave people comfort, meaning and connection, with many regularly attending religious services. While participants in our study reported few worries, good overall personal well-being and were not adversely affected by tragic life events this cohort do appear to draw spiritual comfort from attending religious services and praying during difficult times. This is in accordance with Thompson et al.’s (2020) and Dew et al.’s (2019) qualitative findings which identified that participants placed a high value on spirituality, although very few older adults were represented. These are interesting findings because whether full cognitive function is required to experience spiritual aspects of life has been a matter for debate (Powell 2019, Weathers et al., 2016, Timmins et al., 2016), with some believing that spirituality is not relevant for this group (Barber 2013).

These findings also concur with larger USA studies that find that people with general disabilities (such as mental health issues, physical disabilities, and hearing defects) are much more likely than the general population to rely on prayer as a source of support (Hodge & Reynolds 2019). Indeed, these latter authors term these findings as “unique spiritual and religious profiles” and suggest that it is important to understand “which spiritual and religious dimensions are disproportionately more likely to exist among a given population with a particular disability” (Hodge & Reynolds 2019:75). Similarly, people with physical disabilities are more likely to find comfort from faith that helps them face their challenges (Mugeere et al 2020). While there is little information about spirituality from the perspective of those with intellectual disability, interviews with adults with autism who were non-verbal (using assistive communication technology) revealed a great sensitivity towards their personal spirituality, including strong evidence of transcendental experiences that provided support and hope to individuals (Hills et al 2019). They also expressed a desire to have a life purpose and meaningfully contribute to society (Hills et al 2019).

Expressions of spiritual or religious practices also serve to support self-identity, potential for friendships and opportunities to engage in community activities for this group (Sango and Forrester-Jones 2017). However almost half of the participants in this study indicated difficulty participating in social activities outside of their home, which represented a barrier for engagement in procedural spiritual practice. This is noteworthy as it was found those who attended church or religious groups were more likely to receive comfort.

Barber (2011) identified anecdotal barriers to people with intellectual disability expressing their spirituality such as lack of support for minority faiths and negative staff attitudes. Although at the same time, from some carers’ perspective, spirituality is perceived as important for young people and adults with intellectual disability in their care, who can receive comfort and enjoyment particularly from faith rituals (Panicker, & Ramesh 2019). Indeed one recent psychosocial intervention for mostly older people with intellectual disability and dementia includes “spiritual reminiscence” (Watchman et al. 2020:1). Similarly, Ouellette-Kuntz et al. (2019) developed a consensus statement on how best to support individuals with intellectual disability as they become frail, revealing spiritual and emotional support as important.

As these are new findings, and little research exists on the topic, it is unclear whether the benefit is due to true engagement with something transcendent, connection with others or due to the social support such activities might provide. However, with regard to religious and spiritual practices, a person with an intellectual disability can engage in these activities at a societal level, without being measured on their achievements. As such, religious / spiritual practices may be a sustainable milieu of social engagement, social cohesion and personal practice for people with intellectual disability across the life span. It is possible therefore that religion and spirituality are required less for transcendence, supportive and coping functions and more for the social support and connectedness. This reflects current understandings of spirituality which are related to connectedness with self, others, the significant and/or the sacred (Kleiven et al., 2021). The importance of connectedness was demonstrated in this study’s findings where participants indicated talking to staff provided the greatest sense of comfort, followed by talking to family and friends. Thus there is potential for spiritual comfort from such practices that foster social support and connectedness, which is in line with Büssing’s (2021) understanding of meeting spiritual care needs by listening to a person and acknowledging their underlying spirituality, whatever this may mean to them are supported. Spiritual practices also serve a social mechanism in society and serve to support cultural rituals. Indeed, there are increasing moves internationally towards a *cultural* religion; this means retaining historical and cultural faith traditions in the context of non-belief, even in highly secular countries (Zuckerman 2009).

Another possible benefit of inclusion in spiritual and indeed social religious practices for those with intellectual disability is that it is one of the few areas of life that has not been ordered into a formal meritocracy. While achievements have become fundamental and essential in all aspects of life, including education, sport, work, and music (Young 1994); church involvement provides social engagement and cohesion without the necessity for attainment. Thus the decline of wide scale religious practice and engagement may have a disproportionate impact on this cohort as there are significant barriers to them participating in other areas of contemporary culture which have become increasingly focused on measurement of advancement and attainment. Furthermore, despite great societal advances Western society can serve to exclude people with disability who cannot partake fully in advancement, achievement and acceleration. Contrast this for example with the Navajo, whose traditional family values and roles provides for empowerment of those with disability and full integration within the large, extended family unit (Kapp 2011).

What is not clear from the findings is the extent to which spiritual care or religious practices are influenced by family or carers. Staff working with people with intellectual disability certainly support spiritual needs (Powell 2019) and have been found to support attendance at religious services (Sango and Forrester-Jones 2017). However these supports are usually relished by participants who are “eager to attend” often “waking up early” with excitement (Sango and Forrester-Jones 2017:288). However at the same time, Sango and Forrester-Jones (2014) caution against widespread adoption of supporting attendance at religious services as formal religious approaches can be quite exclusionary (Panicker & Ramesh 2019, Sango and Forrester-Jones 2017, Sheerin, 2013). This cohort are also often highly reliant on others for their social activity and caution also needs to be taken so that religious preferences reflect the needs of the person with intellectual disability rather than their carer or organisation (Kirkendall et al., 2017). Carers need to be aware of meanings of spirituality, comfortable with their own spirituality; and aware of how to provide spiritual support (Barber 2013). At the same time participation in community activities such as religious services is viewed as very important by older people with intellectual disability (Carter et al 2017). These authors recommend this form of engagement in terms of inclusivity and what can be gained from this connection. Conversely Sango and Forrester-Jones’s (2017) review revealed that staff shared that clients with intellectual disability were often treated disrespectfully at religious services (due to disrupting proceedings for example). However Sango and Forrester-Jones (2019) did find that staff working with people with intellectual disability provided different types of support, depending on the organisation. In their experience faith based organisations were more likely to offer non-religious support in addition to faith based support, whereas more secular organisations opted for a more ritualistic approach to spiritual care through the provision of religious supports (Sango and Forrester-Jones 2019).

Despite research findings that many people with intellectual disability find spirituality important (Turner et al., 2004) significant gaps exist at both a policy (Sango and Forrester-Jones 2014) and practice level (Barber 2013) with regard to effectively supporting spiritual needs. Where people with intellectual disability are supported within formalised health and social care structures, knowledge and information is needed about how best to do this (Barber 2013). Recent emerging guidelines for nurses provide good guidance for this including the need for self-awareness, assessment, intervention and evaluation (EPICC 2021) however more needs to be done in terms of raising societal awareness related to spiritual support but also full integration into community faith supports.

Additionally the requirement to effectively manage end of life care for people with intellectual disability is becoming increasingly important (McCallion et al., 2017) and spirituality and spiritual care are essential components of this (Voss et al., 2020). However spirituality and spiritual support has more far reaching implications that simply end of life care. The necessity, requirement and benefit of spirituality and/or attendance at faith based services for those older adults with intellectual disability needs further investigation, along with the competencies and skills required by support workers and families to provide the best support in this context

## Limitations and future research

Although IDS-TILDA is supported by objective measurement this research relies on self-report. At the same time there are challenges to objective measurement of religious and spiritual practices, and the strength of the robust sampling method and valid, reliable approach to data collection within the context of a national longitudinal study, strengthens the validity and transferability of the findings. It is also acknowledged that the data were collected within a specific cultural context.

## Conclusion

IDS-TILDA is influencing policy and practice change both in the ROI and globally (Wormald et al., 2019). There are important patterns and trends emerging in relation to lifestyle that differ greatly from the larger national population. This paper reports for the first time on the religious and spiritual practices of older adults with intellectual disability. It is clear that participants seek and receive solace from religious and spiritual practices, especially if they are lonely, in poor health, distressed or bereaved. There is likely a social benefit to spiritual and religious support that could be highly beneficial to explore within this cohort as loneliness in particular is a challenge for this population. Efforts should be made to improve access and increase opportunities for inclusion in societal spiritual and religious activities where desired and where relevant. Cognisance also needs to be taken automatic inclusion for everyone, particularly in settings where faith based ritual is provided as part of an institutional service. Rather spirituality should be facilitated upon individual request, or spiritual needs assessment. Additional research is required to firmly establish appropriate means of spiritual needs assessment and effective spiritual support of this population.
